# Knowledge, perceptions and media use of the Dutch general public and healthcare workers regarding Ebola, 2014

**DOI:** 10.1186/s12879-017-2906-7

**Published:** 2018-01-08

**Authors:** Lianne G. C. Schol, Madelief Mollers, Corien M. Swaan, Desirée J. M. A. Beaujean, Albert Wong, Aura Timen

**Affiliations:** 1 Centre for Infectious Disease Control, National Institute for Public Health and the Environment (RIVM), PO Box 1 (interne 13), 3720 BA Bilthoven, The Netherlands; 2 Department of Statistics, Informatics and Mathematical Modelling, National Institute for Public Health and the Environment (RIVM), Bilthoven, The Netherlands

**Keywords:** Ebola, Risk perception, Perceived severity, Perceived fear, Perceived susceptibility

## Abstract

**Background:**

The Ebola outbreak in West-Africa triggered risk communication activities to promote adequate preventive behaviour in the Netherlands. Our study investigated the level of knowledge, perceptions, and media use regarding Ebola.

**Methods:**

In December 2014, an online questionnaire was administered to the Dutch population (*n* = 526) and Health Care Workers (HCW) (*n* = 760).

**Results:**

The mean knowledge score (range 0–15) of HCW (m = 13.3;SD = 1.4) was significantly higher than the general public (m = 10.8;SD = 2.0). No significant difference was found in perceived severity and susceptibility. Perceived fear of the general public (m = 2.5; SD = 0.8) was significantly higher than among HCW (m = 2.4; SD = 0.7). Respondents primarily used television to obtain information.

**Conclusions:**

While Ebola was perceived severe, it did not lead to excessive fear or perceived susceptibility for developing the disease. Nonetheless, our research showed that knowledge with respect to human-to-human transmission is low, while this is crucial to complying with preventive measures. Our study reveals priorities for improving risk communication.

**Electronic supplementary material:**

The online version of this article (10.1186/s12879-017-2906-7) contains supplementary material, which is available to authorized users.

## Background

Ebola is characterized by a severe clinical picture and a case-fatality rate of 50–90% in outbreaks in Africa [1]. Ebola virus can be introduced in the human population by direct contact with infected wild animals and is further spread through direct contact with tissues and bodily fluids of infected people [2]. Ebola patients are infectious when symptomatic [3].

Since the onset of the Ebola outbreak in December 2013, WHO has reported 28.610 cases of Ebola, with 11.308 deaths [4]. Guinea, Liberia and Sierra Leone were the three countries with the most widespread and intense transmission of Ebola. Countries that have reported imported cases or secondary transmission of Ebola are Mali, Nigeria, Senegal, the USA, the United Kingdom, Spain and Italy [5]. In August 2014, faced with the likelihood of further international spread and the need for international collaboration to control the outbreak, the WHO declared a Public Health Emergency of International Concern. [6, 7]. WHO has currently declared all countries Ebola-free since April 2016.

The risk of an imported case with Ebola and consequently, the risk of secondary transmission in the Netherlands was considered to be low to very low [5], mainly due to limited travel to the affected regions, but could not be ruled out [5, 8, 9]. Therefore, public health authorities and health care workers (HCW) undertook preparations to detect, investigate and manage suspected Ebola patients through the development and implementation of guidelines and triage protocols [10]. An emphasis was put on personal protective equipment and infection control measures, to increase compliance with safe hospital practices and diminish fear among HCW of becoming infected.

In December 2014, one patient who had acquired Ebola in Liberia was medically evacuated to the Major Incident Hospital in the Netherlands, upon UN request. The medical evacuation of this patient and the Ebola outbreak in general triggered comprehensive coverage by traditional and social media, echoing perceptions and fear of the general public with regard to the possibility of further spread of Ebola to Western countries. To reduce anxiety and to educate the public on the risks associated with Ebola and on the (ir)relevance of certain preventive and control measures, information (e.g. factsheets about Ebola virus and Q&A’s) was provided on the official websites of the National Public Health Institute and public health services, an Ebola-hotline was established and specific messages were issued to HCW to share new ‘facts’ and communicate advised control measures.

The effectiveness of preventive and control measures depend on the behaviour of people and their trust and willingness to adhere to advised measures [11]. Studies on public perception and preventive behaviour during outbreaks of infectious diseases have shown that perceptions influence the attitude and reaction towards advised control measures and related outbreak response [12–16]. Providing accurate and up-to-date information is important to ensure that the public has a realistic sense of vulnerability regarding the risk of infection. In order to promote adequate preventive behaviour, it is therefore important that public health authorities are aware of how Ebola is perceived, whether the provided information is understood and whether the general public and HCW intend to comply with these measures [11, 17]. Evaluating the penetration of public health messages is of value for future communication during public health emergencies.

The aim of the current study was to assess and compare the level of knowledge, perceptions, use of information sources and information needs of the general public and HCW in the Netherlands with regard to Ebola. We hypothesize that the knowledge level about Ebola will be higher among HCW in comparison to the Dutch general public. Additionally, we hypothesize that higher knowledge levels about Ebola will result in lower levels of perceived fear and susceptibility among both respondent groups. The results of the current study will be used to improve risk communication during future public health emergencies.

## Methods

### Study design and population

A cross sectional survey using an online questionnaire was performed among the general public and HCW between 12 and 24 December 2014. Formal ethical approval from a medical ethical committee was not required for this research in the Netherlands [18, 19], since it does not entail subjecting participants to medical treatment or imposing specific rules of conduct on participants.

Members of the general public (aged ≥18) were recruited via a commercial research panel (http://www.flycatcher.eu) that consists of 16,000 members aged ≥12 years. The members are incentivised to participate. The panel has a representative distribution of demographics with regard to the Dutch general public.

HCW involved in the care for (suspected) Ebola patients, i.e. physicians (infectious disease specialists and emergency physicians), nurses and ambulance staff were recruited via their professional associations. On behalf of the researcher, these associations either sent out an e-mail or posted a call in their newsletters, in which the link to the online survey (Formdesk, Innovero Software Solutions B.V) was provided. The obtained information was processed anonymously. The questionnaire responses were received in de-identified form.

### Questionnaire

The questionnaire was based on the constructs of perceived severity and susceptibility from The Health Belief Model and the Protection Motivation Theory [20] and on questionnaires used during previous outbreaks of infectious diseases (e.g. Salmonella, Influenza (H1N1), Q fever and SARS) [12–16].

The questionnaire consisted of 35 questions, divided into three domains: knowledge, perceptions, and information use and needs. Knowledge was assessed by fifteen true/false statements regarding symptoms, modes of transmission, preventive measures, and treatment. Perceptions (i.e. perceived severity, −fear and -susceptibility) were assessed by multiple items with a five-point Likert scale ranked from 1 to 5 (completely disagree, disagree, neutral, agree, and strongly agree) (see Additional file 1: Table S1). In order to compare Ebola-related risk perceptions to other diseases, respondents were asked to indicate their perceived fear and susceptibility with regard to six other diseases. Exposure to information was assessed by asking questions about how often the respondents talked about Ebola with other people and about their passive and active search for information. Information need was assessed by asking the respondents if they would like to receive additional information. If this was the case, follow-up questions were asked about the preferred type of information, sources, and methods of information distribution. The questionnaire was piloted prior to commencement of the study by a convenience sample of 6 persons. To reach 4% precision and a sample which is representative of the Dutch population at least 500 persons had to be included.

### Data analysis

Data from both surveys were analysed separately and combined using SPSS v. 22.0 (SPSS Inc., Chicago, IL, USA) and R v. 3.1.0. Significance was determined at the 5% level (*p*-value ≤0.05).

#### Coding and validating the constructs

Mean scores were computed for the constructs and they were recoded into a new variable with three options: low (score < 2), average (score 2–3) and high (score > 3). The knowledge construct consisted of fifteen statements, for which each correctly answered statement received a score of 1. A sum score was computed for assessing the knowledge level (ranging from 0 to 15, where 0 is no knowledge and 15 is full knowledge), which was then subsequently categorized into three categories: low (score 0–6), average (score 7–10) and high (score 11–15). The variables assessing how often and where the respondents saw, heard or read about the outbreak of Ebola in West Africa were dichotomized and computed into a new variable indicating the use of the various sources (where 0 indicated an use of less than once a week, and 1 indicated an use of once a week or more frequently). In order to assess internal consistency of three intended constructs related to perceptions (i.e. perceived severity, −fear, and -susceptibility), Cronbachs’s alpha was calculated and the cut-off value was set at 0.6. See also [21]. All constructs met this cut-off (see Additional file 1: Table S2).

#### Assessing differences between two respondent groups

Descriptive statistics were performed for the demographics, knowledge, and use of information sources, for each respondent group separately. The Chi-squared test was used to test the statistical significance of group differences in terms of the distribution regarding knowledge, perceived severity, perceived fear, perceived susceptibility, and need for information, and the t-test was used to describe groups differences in terms of the mean.

#### Testing for associations between constructs and its potential predictors

For each combination of construct and respondent groups (general public and HCW) a regression analysis was performed to identify factors significantly associated with the respective construct. The construct perceived severity was not analyzed as a dependent variable due to the fact that the vast majority of both respondent groups perceived Ebola as severe. In total, eight regression models were fitted, of which most concern a construct with three categories (low, medium, high) that was analyzed with ordered logistic regression [22]. Exceptions to this are the model concerning knowledge for the HCW group, which was dichotomized to ‘very high’ and ‘high’ because few had scored low, and the two models concerning need for information (no/yes). These models were analyzed with binary logistic regression.

The following independent variables were included in each model for the general public: sex, age (categorized as 0–25, 26–35, 36–45, 46–55, 56–65, and 65+), nationality (categorized as Dutch, or non-Dutch), children in household (categorized as: children not present, at least one child present), education level (categorized as low, intermediate and high), and working in the healthcare sector (no/yes). The models for HCW included the following independent variables sex, age, nationality, children in household, profession (physician, nurse, ambulance staff), years of working experience (0–1, 2–6, 6–10, and > = 11 years), and weekly worktime spend on topics related to Ebola (no/yes).

## Results

### Demographics

Of the 1286 respondents who returned the questionnaire, 526 (41%) were members of the general public and 760 (59%) were HCW. Except for age ≤ 25, the sample of the general public (response rate of 57%) is representative for the Dutch population. Thirteen percent of the general public worked in the healthcare sector.

The group of HCW consisted of nurses (59%, *n* = 446), physicians (18%, *n* = 136), and ambulance staff (12%, *n* = 92) (see Table 1). The majority of HCW had ≥11 years of working experience (52%, *n* = 397). 47% (*n* = 356) of HCW spend no time, 49% (*n* = 372) spend less than 25% and 4% (*n* = 32) spend more than 25% of their weekly working time on topics related to Ebola.

**Table 1:** Respondent demographics of the Dutch general public and HCW, and *p*-values resulting from a series of Chi-square tests that test for a difference in demographic distribution between general public and HCW for each demographic^a^

Demographic	General public *N* = 526		Healthcare workers *N* = 760		*p*-value
	n	%	n	%	
Sex					
Male	277	52.7	376	49.5	
Female	249	47.3	384	50.5	
Age					*<0.001*
≤ 25	36	6.8	3	0.4	
26 to 35	73	13.9	135	17.8	
36 to 45	90	17.1	233	30.7	
46 to 55	92	17.5	276	36.3	
56 to 65	115	21.9	110	14.5	
> 65	120	22.8	3	0.4	
Parental status					*<0.001*
Single	107	20.3	87	11.4	
Living together or married; with children	143	27.2	452	59.5	
Living together or married; no children	234	44.5	188	24.7	
One-parent family	12	2.3	28	3.7	
Other	30	5.7	5	0.7	
Children in home					*<0.001*
Yes	155	29.5	480	63.2	
No	371	70.5	280	36.8	
Nationality					*<0.001*
Dutch	475	90.3	750	98.7	
Other	51	9.7	10	1.3	
Level of education					*N/T*
Low	176	33.5			
Intermediate	221	42.0			
High	129	24.5			
Works in healthcare sector					*N/T*
Yes	69	13.1			
No	457	86.9			
Profession					*N/T*
Nurse			446	58.7	
Physician			136	17.9	
Ambulance staff			92	12.1	
Other			86	11.3	
Years of working experience					*N/T*
≤ 1			29	3.8	
2 to 5			165	21.7	
6 to 10			169	22.2	
≥ 11			397	52.2	
Amount of worktime spent on topics related to Ebola					*N/T*
No time spent			356	46.8	
≤ 25%			372	48.9	
26 to 50%			18	2.4	
51 to 75%			7	0.9	
> 75%			7	0.9	

^a^If left blank, the demographics only apply to either the general public or HCW; no Chi-square tests were performed for these demographics (as denoted by “N/T”)

Compared to the general public, HCW were younger (*p* < 0.001), were significantly more often Dutch (99%; *n* = 750 vs. 90%;*n* = 475; *p* < 0.001), lived together with a partner more often (84%;*n* = 640 vs. 72%;*n* = 377; *p* < 0.001) and there were more often children in the household (63%;*n* = 480 vs. 29%;*n* = 155; *p* < 0.001) (Table 1).

### Knowledge

The mean knowledge sum score (range 0–15) of HCW (m = 13.25;SD = 1.44) was significantly higher (*p* < 0.001) than the general public’ score (m = 10.84;SD = 21.97) (see Table 2). 62.4% (*n* = 328) of the general public had high levels (sum score of 11 or higher)) of knowledge, which was significantly lower (*p* < 0.001) than for the HCW (95% of 760 individuals) (see Table 2). Ten percent (*n* = 51) of the general public and 32% (*n* = 243) of HCW knew that Ebola cannot be transmitted through coughing. The fact that only symptomatic patients with Ebola are infectious was known by 18% (*n* = 96) of the general public and 66% (*n* = 505) of HCW. HCW scored higher on all statements. This difference was significant for eleven of the fifteen presented statements (see Additional file 1: Table S1).

**Table 2:** Descriptives and statistical significance of knowledge, perceived severity, perceived fear, perceived susceptibility, and need for information of the general public and HCW

	General public *N* = 526		Healthcare workers *N* = 760		*p*-value^c^
	mean or n	SD or %	mean or n	SD or %	
Knowledge					
Mean sum score (SD)	10.84 (1.97)		13.25 (1.44)		*<0.001*
High level of knowledge^a^	328	62.4	*560*	95.1	<0.001
Perceived severity					
Mean score (SD)	4.72 (0.46)		4.74 (0.45)		
High level of perceived severity^b^	516	98.1	*746*	98.2	
Perceived fear					
Mean score (SD)	2.94 (0.79)		2.83 (0.73)		*<0.05*
High level of perceived fear^b^	116	22.1	*121*	15.9	<0.01
Perceived susceptibility					
Mean score (SD)	3.29 (0.65)		3.25 (0.60)		
High level of perceived susceptibility^b^	173	32.9	*244*	32.1	
Need for additional information					
Category Yes	361	68.6	564	74.2	<0.05

^a^High level of knowledge is mean sum score is 11 or higher

^b^High level perceived severity, perceived feelings of concern and perceived susceptibility if scored 4 or 5

^c^Chi-squared test was used to test statistical significance of group differences for levels/categories, *t*-test was used to test for group differences for scores

In the regression analysis, predictors of higher levels of knowledge among the general public were age (46–55 and 56–65 years) and educational level (intermediate and high). Having a household with children was significantly related to lower levels of knowledge (see Table 3).

**Table 3:** Predictors of knowledge, perceived fear, perceived susceptibility and need for information

		Knowledge		Perceived fear		Perceived susceptibility		Need for information	
		GP	HCW	GP	HCW	GP	HCW	GP	HCW
		OR	OR	OR	OR	OR	OR	OR	OR
1	Sex: female	0.95	0.91	1.63*	1.08	1.34	1.17	1.2	0.72
2	Age: 26–35 years	1.72	2.59	0.41*	0.39	0.8	0.78	0.56	<0.01
	Age: 36–45 years	1.84	3.22	0.45	0.33	0.88	0.58	1.08	<0.01
	Age: 46–55 years	2.92*	4.22	0.46	0.41	1.31	0.51	0.58	<0.01
	Age: 56–65 years	2.25*	3.67	0.54	0.31	1.49	0.68	0.58	<0.01
	Age: > 65 years	1.8	>1000	1.17	2.97	0.9	0.68	0.97	<0.01
3	Children in home	0.54**	1.19	0.86	1.39*	1.28	0.97	0.76	0.73
4	Nationality: Dutch	1.62	0.6	0.84	1.46	1.13	3.03	0.76	0.29
5	Level of education: intermediate	1.7*	–	0.86	–	1.2	–	0.7	–
	Level of education: high	4.01***	–	0.58	–	0.7	–	0.97	–
6	Works in healthcare sector	1.17	–	0.94	–	0.7	–	0.76	–
7	Profession: nurse	–	0.22***	–	1.7*	–	2.46***	–	1.34
	Profession: ambulance staff	–	0.12***	–	2.69**	–	2.48**	–	1.31
8	Years of working experience: 2–5	–	0.53	–	1.86	–	1.06	–	0.96
	Years of working experience: 6–10	–	0.63	–	1.43	–	1.46	–	0.63
	Years of working experience: > = 11	–	0.55	–	1.52	–	1.48	–	0.65
9	Worktime spend on topics related to Ebola	–	1.92***	–	1.03	–	0.82	–	0.79
10	Knowledge level: intermediate	–	–	5.31**	–	1.34	–	2.53	–
	Knowledge level: high	–	–	4.14*	0.64**	1.02	0.46***	3.25*	0.82
11	Perceived fear: intermediate	–	–	–	–	–	–	1.88**	1.84**
	Perceived fear: high	–	–	–	–	–	–	4.39***	4.31***
12	Perceived susceptibility: intermediate	–	–	13.87***	2.94***	–	–	0.99	0.81
	Perceived susceptibility: high	–	–	181.27***	21.76***	–	–	2.46*	1.38

Reference categories are 1) sex: male, 2) age: ≤25 years, 3) no children in household, 4) other nationality, 5) level of education: low, 6) not working in healthcare sector, 7) profession: physician, 8) years of working experience: ≤1, 9) no worktime spend on topics related to Ebola, 10) knowledge level: low, 11) perceived fear: low, and 12) perceived susceptibility: low

*p < 0.05, **p < 0.01, ***p < 0.001

- variable not included in regression analysis

HCW who reported spending more worktime on topics related to Ebola had significantly higher levels of knowledge. Nurses and ambulance staff had significantly lower levels of knowledge compared to physicians (see Table 3).

### Perceptions

#### Perceived severity

No significant difference was found between the perceived severity of the general public and HCW (see Table 2). The majority of the general public (98%; *n* = 516) and HCW (98%; *n* = 746) perceived Ebola as a severe disease. The respondents considered Ebola to be as severe a threat for their health as HIV/AIDS or a heart attack (see Additional file 1: Table S3).

#### Perceived fear

The perceived fear for Ebola was significantly higher among the general public than HCW (see Table 2). A minority of the general public (22%;*n* = 116) and HCW (16%;*n* = 121) had high levels (mean score of 4 or 5) of perceived fear.

In the regression analysis, predictors of higher levels of perceived fear among the general public were sex (female), knowledge level (intermediate and high), and perceived susceptibility (intermediate and high). Age between 26 and 35 years was significantly related to lower levels of perceived fear compared to other age groups. Perceived susceptibility is of greatest influence on the level of fear (see Table 3).

Predictors of higher levels of perceived fear among HCW were having children at home, profession (nurse and ambulance staff) and perceived susceptibility (intermediate and high). High level of knowledge was significantly related to lower levels of fear. Perceived susceptibility is of greatest influence on the level of fear (see Table 3).

#### Perceived susceptibility

No significant difference was found between the perceived susceptibility to Ebola of the general public and HCW (see Table 2). 33% (*n* = 173) of the general public and 32% (*n* = 244) of HCW had high levels (mean score of 4 or 5) of perceived susceptibility. Of all statements, both respondent groups considered it most likely that an infected healthcare worker might be coming to the Netherlands from abroad, rather than being diagnosed with Ebola themselves or the onset of an Ebola outbreak in the Netherlands (see Additional file 1: Table S2).

Both respondent groups indicated to be most susceptible to Influenza and least susceptible to HIV/AIDS (see Additional file 1: Table S4). The perceived susceptibility of the general public with regard to all diseases (except Salmonella, which was similar) was significantly higher than the perceived susceptibility of HCW.

In the regression analysis, there were no significant predictors of the level of perceived susceptibility among the general public (see Table 3).

The predictor of higher levels of perceived susceptibility among HCW was profession (nurses and ambulance staff). High level of knowledge was significantly related to lower levels of perceived susceptibility. Being ambulance staff is of greatest influence on the level of perceived susceptibility (see Table 3).

### Exposure to- and need for information

The additional need for information of HCW (m = 0.74;SD = 0.44) was significantly higher than the general publics’ additional need for information (m = 0.69;SD = 0.46) (see Table 2). Both groups acknowledged to have been primarily exposed to information through television (general public 90%;*n* = 474 vs HCW 91%;*n* = 694) (see Additional file 1: Table S5).

The general public wished to receive more factual disease-related information (e.g. about symptoms (30%), mode of transmission (23%), and infection prevention (21%). HCW were interested in preventive measures and behavioural information (e.g. about what to do when in contact with Ebola patient (34%), what the Dutch authorities are doing to diminish the risk of importation and spread of Ebola (30%), and how to treat Ebola (22%)). The preferred source of information for the general public was the government (73%). The preferred sources of information for HCW were their employer (45%) or the National Institute of Public Health 38%).

In the regression analysis, predictors of the need for information among the general public were the level of knowledge (high), perceived fear (intermediate and high), and susceptibility (high). A high level of perceived fear is of greatest influence on the need for information (see Table 3).

The predictor of the need for information among HCW was perceived fear (intermediate and high). A high level of perceived fear is of greatest influence on the need for information (see Table 3).

## Discussion

This study evaluates and compares the knowledge, perceptions, use of information sources and information needs of the general public and HCW regarding Ebola in the Netherlands. In brief, the majority of the general public and HCW have some overall knowledge of Ebola, but the knowledge with respect to person-to-person transmission is not optimal. Despite the broad media coverage and the fact that Ebola is perceived as a severe disease, the Dutch general public and HCW appear to have low levels of perceived fear and perceived susceptibility. The respondents report to have been exposed to information primarily from television. The majority of the respondents expressed an additional need for information.

### Knowledge

Despite the fact that Dutch HCW appear to be significantly more knowledgeable than the Dutch general public, this study indicates that both respondent groups have high knowledge levels. Physicians have significantly higher level of knowledge in comparison to nurses and ambulance staff. Nonetheless, knowledge regarding human-to-human transmission of Ebola is low, while this is crucial to the understanding of and compliance with preventive measures. The results of the current study are in line with studies conducted in the same time period in Australia, Germany and Sudan [23–25], also showing that knowledge about modes of transmission is often poor. A common misperception is that Ebola virus spreads through air, Lacking knowledge regarding the transmission route of Ebola (and other diseases with similar severity and propensity to spread) is pivotal, as adequate knowledge provides the basis for the general public, risk groups and HCW to understand the rationale for the measures national authorities take. Insufficient knowledge may cause misunderstandings and anxiety, especially with respect to travellers and HCW, who return from affected areas and need to be able to have access to public facilities in daily life [26]. This is the reason why the control measures and their communication strategies are comprehensively described in disease specific guidelines and national preparedness plans [27].

The Dutch authorities have adopted the policy to actively monitor temperature in high risk contacts instead of applying home quarantine, which restricts the individual freedom of movement. Healthy people *(*i.e. people without any symptoms related to Ebola) returning from affected areas do not pose a threat to public health and quarantine measures are therefore irrelevant. To understand and endorse the measures, appropriate knowledge about Ebola transmission is needed.

We observed a discrepancy with regard to knowledge among the two study groups. As we expected, a higher level of knowledge among HCW is significantly associated with lower levels of perceived fear and lower levels of perceived susceptibility. On the contrary however, having intermediate or high levels of knowledge was significantly related to higher levels of perceived fear and a need for additional information of the general public. These results thus suggest two important aspects: 1) while authorities believe it is important that the general public has (some level of) knowledge with regard to Ebola in order to promote adequate preventive behaviour, this may unintentionally lead to higher levels of perceived fear and an additional need for information, and 2) HCW appear to benefit from higher levels of knowledge. These findings highlight the relevance of specifically tailoring the information to pre-defined target groups and enhancing the relevance of the information presented [26, 28]. They also show that authorities and communication specialists need to focus their efforts in maintaining the delicate balance between too much and just enough information. Studies of knowledge and perceptions during ongoing outbreaks or threats allow for timely readjustment of the information.

### Perceptions

The Dutch general public and HCW perceived Ebola as a severe disease, comparable with HIV/AIDS or a heart attack, perceptions similar to results obtained from studies conducted in other countries [24, 25, 29]. Although perceived severity was high, the perceived susceptibility to Ebola and perceived fear of Ebola among both groups was low. However, when specifically looking at the different HCW, our results show that nurses and ambulance staff have a significantly higher level of perceived fear and perceived susceptibility in comparison to physicians. One possible explanation for this difference may be related to the fact that nurses and ambulance staff are likely to be the first ones to get in contact with a (suspected) Ebola patient and generally have more physical contact with the patient. The results are in line with a study conducted in Germany, in the same time period that revealed similar levels of perceived susceptibility and fear among their participations; 29% of the participants were worried [24].

However, on the contrary, a similar study in the U.S conducted in the same time period, contradicts the traditional notions of health behaviour, as they show that while the perceived susceptibility is low, the behavioural intention (i.e. avoiding public transportation, avoiding those who travelled to affected areas, or changing hygiene practices) is high [29].

Our findings, and the results of similar studies, underline the need to sufficiently educate the public and HCW and to tailor education, training and communication efforts to these specific groups.

### Need for information

The general public and HCW were well aware of the Ebola outbreak in West Africa. The respondents obtained their information primarily from television and newspapers, a finding in accordance with previous outbreaks [12, 15, 30–32] and similar studies [23–25]. The official websites of the National Public Health Institute and public health services, and the dedicated Ebola-hotline were less often consulted than traditional media to gather information. However, these results may be subject to recall bias. When it comes to preferred and trusted information channels, the government, the National Public Health Institute, and the employer were mentioned.

### Strengths and limitations

A strength of this study is the comparison, at the same point in time, of two respondent groups; the general public and three groups of HCW. Especially since we show that the level of knowledge, perceptions, exposure to information sources and information needs of the general public and HCW varies. Another strength of this study is that a large proportion of the included HCW were nurses. Nurses are closely involved in the care for infectious patients and are at higher risk for acquiring an infection (for secondary transmission), highlighting the relevance of assessing their level of risk perception.

Our study has several limitations. This is a cross-sectional survey and thus trends over time could not be identified due to the design of this study. Our study was restricted to the Dutch general public and a selection of Dutch HCW. We cannot state with certainty that our results are also applicable to other (Western) countries.

## Conclusions

We have shown that the Ebola outbreak in West Africa has not led to excessive fear or perceived susceptibility for developing the disease among the Dutch population and HCW, despite the fact that the disease Ebola was perceived as (very) severe and the study was undertaken immediately after the medical evacuation of a patient with confirmed Ebola to the Netherlands. The results of the current study indicated that the Dutch population and HCW were well aware of the Ebola outbreak in West Africa and were exposed to information primarily via traditional media (i.e. television and newspapers). The majority of the Dutch general public and HCW had an additional need for information. The general public more often expressed a need for factual disease-related information; HCW more often expressed a need for information on personal protection and preventive measures. Despite the lack of specific knowledge with regard to the transmission of Ebola, the overall level of knowledge among the respondents was high.

The results of the study can be used to improve risk communication during future outbreaks and threats. The importance of specifically tailoring information and education to the different groups of HCW is underpinned by our results. Since this study has been carried out rather early during the course of the Ebola outbreak in West Africa, the results have been directly communicated to the by the communication experts allowing them to specifically address the information needs about Ebola transmission. This has been the most valuable lesson learned following the execution of the study, with clear policy implications related to the content and preferred channels for information provision to HCW and public. Future research should focus on assessing the level of knowledge, risk perception and information needs of the general public and HCW at different points in time as the epidemic unfolds, to allow for re-adjustments of the communication of risks and preventive measures.

## Additional file

Additional file 1: Table S1. Knowledge about Ebola among Dutch general public and healthcare workers. **Table S2.** Mean scores and Cronbach’s alpha of items in three constructs (perceived severity, perceived susceptibility, and perceived fear). **Table S3.** Populations’ estimation of severity of various diseases. **Table S4.** Populations’ estimation of susceptibility to various (infectious) diseases. **Table S5.** Respondents’ information intake per source (*n* = 1286). (DOCX 26 kb)
