# Supplementary material for: Knowledge, perceptions and media use of the Dutch general public and healthcare workers regarding Ebola, 2014
Source: BMC Infect Dis. 2018 Jan 8;18:18. doi: 10.1186/s12879-017-2906-7 (PMC5759181; doi:10.1186/s12879-017-2906-7)
Supplement: Additional file 1: Table S1. — Knowledge about Ebola among Dutch general public and healthcare workers. Table S2. Mean scores and Cronbach’s alpha of items in three constructs (perceived severity, perceived susceptibility, and perceived fear). Table S3. Populations’ estimation of severity of various diseases. Table S4. Populations’ estimation of susceptibility to various (infectious) diseases. Table S5. Respondents’ information intake per source (n = 1286). (DOCX 26 kb) [file 12879_2017_2906_MOESM1_ESM.docx]

**Additional file**

Table S1: Knowledge about Ebola among Dutch general public and healthcare workers

| **Statement (correct answer)** | **General public**  **N = 526** | | **Healthcare**  **workers N = 760** | | **OR (95% CI)** |
| --- | --- | --- | --- | --- | --- |
|  | **n** | **%** | **n** | **%** |  |
| 1. Ebola is caused by a virus (true) | 449 | 85.4 | 734 | 96.6 | 0.21 (0.13-0.33)*** |
| 2. Ebola is spread in the human population through human-to-human transmission (true) | 519 | 98.7 | 751 | 98.8 | 0.89 (0.33-2.40) |
| 3. Ebola can be transmitted through coughing (false) | 51 | 9.7 | 243 | 32.0 | 0.23 (0.17-0.32)*** |
| 4. Ebola can be spread through killing and eating of a sick animal (bushmeat) in Africa (true) | 205 | 39.0 | 610 | 80.3 | 0.16 (0.12-0.20)*** |
| 5. People infected with Ebola are also contagious when they do not show any symptoms (false) | 96 | 18.3 | 505 | 66.4 | 0.13 (0.09-0.15)*** |
| 6. A registered vaccine against Ebola is available (false) | 342 | 65.0 | 680 | 89.5 | 0.22 (0.16-0.29)*** |
| 7. In order to prevent spreading of Ebola, rapidly recognizing patients is necessary (true) | 518 | 98.5 | 752 | 98.9 | 0.69 (0.26-1.85) |
| 8. Isolating patients is useless in order to control the Ebola spread (false) | 473 | 89.9 | 731 | 96.2 | 0.35 (0.22-0.57)*** |
| 9. Bleeding can be a symptom of Ebola (true) | 300 | 57.0 | 689 | 90.7 | 0.14 (0.10-0.18)*** |
| 10. Hygiene measures are useless to control spread of Ebola(false) | 464 | 88.2 | 735 | 96.7 | 0.26 (0.16-0.41)*** |
| 11. Someone who has questions about Ebola can contact the Municipal Health Services (GGD) (true) | 444 | 84.4 | 714 | 93.9 | 0.35 (0.24-0.51)*** |
| 12. Someone who has been in contact with a (suspected) Ebola patient and who develops fever within 21 days, should contact their general practitioner (true) | 505 | 96.0 | 733 | 96.4 | 0.89 (0.50-1.58) |
| 13. Ebola is a common disease in the Netherlands (false) | 522 | 99.2 | 758 | 99.7 | 0.34 (0.06-1.89) |
| 14. Ebola can be detected through blood tests (true) | 331 | 62.9 | 692 | 91.1 | 0.17 (0.12-0.23)*** |
| 15. Wearing personal protective equipment is useless in order to prevent infection with Ebola (false) | 484 | 92.0 | 740 | 97.4 | 0.31 (0.18-0.54)*** |
| * p < 0.05, ** p < 0.01, *** p < 0.001 |  |  |  |  |  |

Table S2: Mean scores and Cronbach’s alpha of items in three constructs (perceived severity, perceived susceptibility, and perceived fear)

|  | **General public**  **N = 526** | | **Healthcare workers N = 760** | |
| --- | --- | --- | --- | --- |
|  | **Mean (SD)** | **Cronbach’s alpha** | **Mean (SD)** | **Cronbach’s alpha** |
| **Perceived severity** |  | *0.825* |  | *0.823* |
| How serious do you think Ebola is? | 4.63 (0.58) | 0.908 | 4.62 (0.58) |  |
| How awful would it be if you would be diagnosed with Ebola? | 4.77 (0.53) | 0.716 | 4.83 (0.48) |  |
| How awful would it be if someone in your vicinity would be diagnosed with Ebola? | 4.76 (0.51) | 0.639 | 4.78 (0.51) |  |
| **Perceived susceptibility** |  | *0.620* |  | *0.619* |
| How likely is it that someone infected with Ebola will be coming to the Netherlands in the upcoming year? | 3.83 (0.88) | 0.562 | 4.04 (0.82) |  |
| How likely is it that a healthcare worker with Ebola will be coming to the Netherlands in the upcoming year? | 3.91 (0.79) | 0.558 | 4.08 (0.77) |  |
| How likely is the onset of an Ebola outbreak in the Netherlands in the upcoming year? | 2.20 (0.88) | 0.508 | 1.89 (0.82) |  |
| How likely is it that you will be diagnoses with Ebola in the upcoming year? | 1.77 (0.82) | 0.570 | 1.63 (0.72) |  |
| **Perceived fear** |  | *0.885* |  | *0.843* |
| How worried are you about Ebola at this moment? | 3.06 (1.14) | 0.872 | 2.93 (1.09) |  |
| How afraid of Ebola are you at this moment? | 2.46 (0.99) | 0.854 | 2.32 (0.92) |  |
| How often do you think about Ebola at this moment? | 2.16 (0.86) | 0.892 | 2.48 (0.91) |  |
| How afraid are you that someone infected with Ebola will be coming to the Netherlands in the upcoming year? | 2.84 (0.98) | 0.860 | 2.61 (0.97) |  |
| How afraid are you that a healthcare worker infected with Ebola will be coming to the Netherlands in the upcoming year? | 2.96 (1.02) | 0.866 | 2.70 (1.04) |  |
| How afraid are you for the onset of an Ebola outbreak in the Netherland in the upcoming year? | 2.29 (0.96) | 0.861 | 2.00 (0.87) |  |
| How afraid are you that you will be diagnoses with Ebola in the upcoming year? | 1.94 (0.90) | 0.872 | 1.83 (0.84) |  |

Table S3: Populations' estimation of severity of various diseases

|  | **General public**  **N = 526**  **Mean (SD)** | **HCW N = 760**  **Mean (SD)** | **p-value** |
| --- | --- | --- | --- |
| **Ebola** | 4.81 (0.49) | 4.84 (0.49) | 0.236 |
| **HIV/AIDS** | 4.76 (0.51) | 4.62 (0.57) | *<0.001* |
| **Heart attack** | 4.74 (0.51) | 4.63 (0.55) | *<0.001* |
| **Diabetes mellitus** | 4.17 (0.73) | 4.07 (0.67) | *0.013* |
| **Salmonella infection** | 4.05 (0.80) | 3.38 (0.91) | *<0.001* |
| **Asthma** | 4.04 (0.72) | 3.76 (0.76) | *<0.001* |
| **Influenza** | 2.48 (1.00) | 2.04 (0.90) | *<0.001* |

Table S4: Populations' estimation of susceptibility to various (infectious) diseases

|  | **General public**  **N = 526**  **Mean (SD)** | **HCW N = 760**  **Mean (SD)** | **p-value** |
| --- | --- | --- | --- |
| **Ebola** | 1.71 (0.78) | 1.49 (0.68) | *<0.001* |
| **HIV/AIDS** | 1.46 (0.70) | 1.36 (0.56) | *0.006* |
| **Heart attack** | 2.29 (0.87) | 1.95 (0.76) | *<0.001* |
| **Diabetes mellitus** | 1.98 (0.96) | 1.74 (0.71) | *<0.001* |
| **Salmonella infection** | 2.29 (0.76) | 2.29 (0.75) | 0.949 |
| **Asthma** | 1.93 (0.93) | 1.67 (0.81) | *<0.001* |
| **Influenza** | 3.52 (1.02) | 3.39 (0.99) | *0.031* |

Table S5: Respondents' information intake per source (n = 1286)^a^

|  | **General public**  **N = 526** | | **Healthcare**  **workers N = 760** | | **OR (95% CI)** |
| --- | --- | --- | --- | --- | --- |
|  | **n** | **%** | **n** | **%** |  |
| **Television** | 474 | 90.1 | 694 | 91.3 | 0.87 (0.59-1.27) |
| **Newspaper** | 362 | 68.8 | 609 | 80.1 | 0.55 (0.42-0.71)*** |
| **Internet** | 331 | 62.9 | 627 | 82.5 | 0.36 (0.28-0.47)*** |
| **Current event websites** | 354 | 67.3 | 599 | 78.8 | 0.55 (0.43-0.71)*** |
| **Radio** | 319 | 60.6 | 580 | 76.3 | 0.48 (0.38-0.61)*** |
| **Newspaper websites** | 278 | 52.9 | 377 | 49.6 | 1.14 (0.91-1.42) |
| **Facebook** | 115 | 21.9 | 127 | 16.7 | 1.40 (1.05-1.85)* |
| **Twitter** | 52 | 9.9 | 96 | 12.6 | 0.76 (0.53-1.09) |
| **Forums/blogs** | 41 | 7.8 | 66 | 8.7 | 0.89 (0.59-1.34) |
| **Ebola-information point** | 25 | 4.8 | 77 | 10.1 | 0.44 (0.28-0.71)*** |
| **GGD** | 24 | 4.6 | 177 | 23.3 | 0.16 (0.10-0.25)*** |
| **RIVM website** | 21 | 4.0 | 182 | 23.9 | 0.13 (0.08-0.21)*** |
| ^a^ Use of information source once a week or more frequently  * p < 0.05, ** p < 0.01, *** p < 0.001 | | | | | |
